# Successful Conservative Management After Expulsion Following Fetal Demise in a Triplet Pregnancy: A Case Report

**DOI:** 10.7759/cureus.102153

**Published:** 2026-01-23

**Authors:** Farah Karam, Nour Jaalouk, Joseph Azoury

**Affiliations:** 1 Medical School, University of Balamand (UOB), Balamand, LBN; 2 Obstetrics and Gynecology, University of Balamand (UOB), Balamand, LBN; 3 Obstetrics and Gynecology, Mount Lebanon Hospital University Medical Center, Hazmieh, LBN

**Keywords:** assisted reproductive technology (art), conservative medical management, preterm premature rupture of membranes, selective fetal loss, triplet pregnancy

## Abstract

Triplet pregnancies are associated with high rates of complications, including miscarriage, preterm birth, and selective fetal loss. We present a case of a 42-year-old Caucasian woman with a history of infertility, multiple failed in vitro fertilization (IVF) attempts, and one prior pregnancy loss at 26 weeks of gestation due to placenta previa. She conceived trichorionic triamniotic (TCTA) triplets following intracytoplasmic sperm injection (ICSI) with donor oocytes. At 18 weeks and five days, she developed preterm premature rupture of membranes (PPROM) with umbilical cord prolapse of one fetus, which already had no detectable fetal heart activity. Intrauterine cord ligation (IUCL) was performed, and the demised fetus was retained until expulsion the following day. Conservative management with progesterone support, antibiotics, anticoagulation, tocolysis with atosiban (OXTR/V1A oxytocin and vasopressin V1A receptor antagonist), and antihypertensive therapy allowed prolongation of the pregnancy. Despite challenges including intrauterine growth restriction (IUGR), hypertension, hypothyroidism, and anemia, the remaining two fetuses continued to develop with reassuring Doppler ultrasonography (DUS) findings. At 33 weeks and two days, she underwent urgent cesarean delivery of two viable preterm infants for non-reassuring fetal heart rates (FHRs).

This case highlights that with individualized management and intensive monitoring, continuation of pregnancy after selective fetal loss in a triplet gestation is possible, improving neonatal outcomes.

## Introduction

The incidence of multifetal pregnancies has risen significantly in recent decades, largely due to the widespread use of assisted reproductive technologies (ART) and delayed maternal age at conception. Triplet pregnancies account for approximately 0.1-0.2% of all pregnancies, and despite improved embryo transfer policies, they continue to occur and remain associated with considerable maternal and perinatal risks [1]. Compared with singleton pregnancies, triplet gestations carry a markedly higher risk of preterm birth, perinatal mortality, and neonatal morbidity, as well as increased maternal complications [2].

The physiological strain of carrying three fetuses results in greater uterine distension, increased placental mass, and heightened metabolic demands, all of which predispose to obstetric complications. Maternal risks include miscarriage, hypertensive disorders of pregnancy (HDP), gestational diabetes mellitus (GDM), anemia, preeclampsia (PE), postpartum hemorrhage (PPH), and a higher likelihood of cesarean delivery. Despite advances in prenatal and neonatal care, perinatal mortality and morbidity rates in triplet pregnancies remain substantially higher than those observed in singleton and twin gestations [3].

An important determinant of outcomes in multifetal pregnancies is chorionicity and amnionicity, which influence fetal growth, inter-fetal vascular connections, and the risk of complications. Triplet pregnancies may be classified as trichorionic triamniotic (TCTA), dichorionic triamniotic, or monochorionic configurations, with TCTA pregnancies generally associated with lower risks of inter-fetal complications compared to monochorionic gestations.

Selective fetal reduction (SFR) or spontaneous selective fetal demise (sSFD) represents an additional clinical challenge in multifetal pregnancies. Such events may occur spontaneously due to unequal placentation or cord accidents, or iatrogenically following invasive procedures [4]. The prognosis for the surviving fetuses largely depends on gestational age at loss and placental architecture, particularly the presence or absence of shared circulation.

Optimal management following selective fetal loss in triplet pregnancies remains poorly defined, with limited evidence and no clear consensus regarding conservative versus interventional approaches. While immediate uterine evacuation may be indicated in cases of infection or maternal compromise, conservative management can be a viable option when maternal and fetal conditions are stable, allowing prolongation of gestation and improved neonatal outcomes while minimizing maternal morbidity [5].

This report presents a rare case of a TCTA triplet pregnancy complicated by selective fetal loss, highlighting the role of chorionicity, multidisciplinary decision-making, and conservative management in achieving a favorable outcome.

## Case presentation

Patient profile

A 42-year-old Caucasian woman (blood type O positive) presented with a multifetal pregnancy. Her husband’s blood type was B positive. She had a long-standing history of primary infertility, attributed to poor embryo quality and her husband’s severe oligospermia (7 million sperm/mL, 0% motility). She had undergone more than 20 failed in vitro fertilization (IVF) cycles, including multiple attempts with donor oocytes. Her past medical history included hypothyroidism controlled with levothyroxine, and her obstetrical history was significant for one pregnancy loss at 26 weeks of gestation due to placenta previa. She was immune to Toxoplasma, cytomegalovirus (CMV), and rubella.

She conceived a triplet pregnancy on January 22, 2025, following intracytoplasmic sperm injection (ICSI) with donor oocytes; three embryos were transferred after her informed consent. The estimated due date was October 15, 2025.

Early pregnancy course

From the day of transfer, the patient received estradiol, vaginal progesterone, short-term prednisone, aspirin, vitamin D (VitD), folate, and a one-week course of azithromycin. Progesterone levels on day 7 post-transfer were 21 ng/mL.

On February 7 (four weeks and two days), 2025, beta-human chorionic gonadotropin (β-hCG) was 1135 mIU/mL, rising appropriately to 3101.43 mIU/mL on February 9. Estradiol was 564 pg/mL, and progesterone was 13.1 ng/mL. Progesterone support was continued at 25 mg daily.

Spotting occurred on February 17 (five weeks and five days), prompting reinforcement of progesterone support. On February 21 (six weeks and two days), mild bleeding led to ultrasound confirmation of a triplet pregnancy with three positive fetal heart rates (FHRs), three yolk sacs, and three fetal poles. Blood pressure (BP) was 130/90 mmHg, and weight was 52 kg. Antihypertensive therapy with methyldopa was initiated, and hypothyroidism was controlled with levothyroxine.

Subsequent laboratory evaluation showed normal glucose levels, adequate VitD status, and negative urine analysis and culture. Serial ultrasounds up to 15 weeks confirmed appropriate early growth, stable cervical length, and reassuring fetal cardiac activity in all three fetuses.

Fetal loss and conservative management

On May 20, 2025 (18 weeks and five days), the patient presented with leakage of fluid. Examination revealed a prolapsed umbilical cord of one fetus protruding through the vagina, with no detectable FHR, confirming fetal demise.

Given that the umbilical cord was clearly visible vaginally, cord ligation was performed via the vaginal route, without the need for transabdominal or ultrasound-guided intervention. The exposed cord was clamped, ligated using absorbable surgical sutures (Vicryl), cut, and carefully reinserted into the uterine cavity to minimize bleeding and reduce the risk of ascending infection. The demised fetus was intentionally left in situ, and spontaneous expulsion of the previable fetus with its placenta occurred naturally the following day. No cerclage was placed due to the increased risk of infection.

Tocolysis with atosiban was administered in two cycles (26 May-30 May and 31 May-8 June 2025) following standard dosing protocols. The use of atosiban allowed suppression of uterine activity after expulsion of the demised fetus, preventing progression to preterm labor and enabling continuation of the pregnancy for the remaining fetuses.

Conservative management included broad-spectrum intravenous antibiotics, continued progesterone support, methyldopa, low-molecular-weight heparin (LMWH, Lovenox 40 mg daily), and strict bed rest. Maternal monitoring consisted of daily clinical assessment and serial laboratory testing, including complete blood count, inflammatory markers (C-reactive protein (CRP), procalcitonin), coagulation profile, electrolytes, and renal and liver function tests, to detect early signs of infection, coagulopathy, or maternal compromise.

Laboratory trends showed anemia, transient inflammatory marker elevation, electrolyte disturbances, and reactive thrombocytosis, all of which gradually normalized with conservative management. Vaginal cultures remained negative throughout hospitalization.

Pregnancy progression

Serial ultrasounds were performed to monitor fetal growth, Doppler indices, and amniotic fluid volumes. Fetal growth parameters are summarized in Table 1, demonstrating progressive weight gain in both surviving fetuses despite early growth restriction. Doppler studies showed preserved end-diastolic flow, and amniotic fluid volumes remained within normal limits.

**Table 1:** Serial ultrasound assessment of fetal growth in the two surviving fetuses following selective fetal loss CPI: cerebroplacental index; EDF: end-diastolic flow; AFI: amniotic fluid index

Gestational Age	Fetus A Estimated Weight (g)	Fetus B Estimated Weight (g)	Presentation / Notes
24 + 2 weeks	563 g (4th percentile)	495 g (<1st percentile)	Fetus A breech; Fetus B cephalic; abnormal CPI in Fetus B with preserved EDF
25 weeks	618 g	587 g	Continued growth
26 + 3 weeks	708 g	638 g	Stable Dopplers
27 + 2 weeks	820 g	670 g	Normal AFI
28 + 2 weeks	820 g	770 g	Interval growth
29 + 2 weeks	930 g	850 g	Ongoing surveillance
31 + 2 weeks	1270 g	1110 g	Cervical shortening noted
32 + 6 weeks	1635 g	1440 g	Pre-delivery assessment

Cervical length shortened gradually in the third trimester. Episodes of spotting and reduced fetal movements prompted close surveillance and hospital admission at 33 weeks and one day.

Delivery and peripartum management

**Figure 1:**
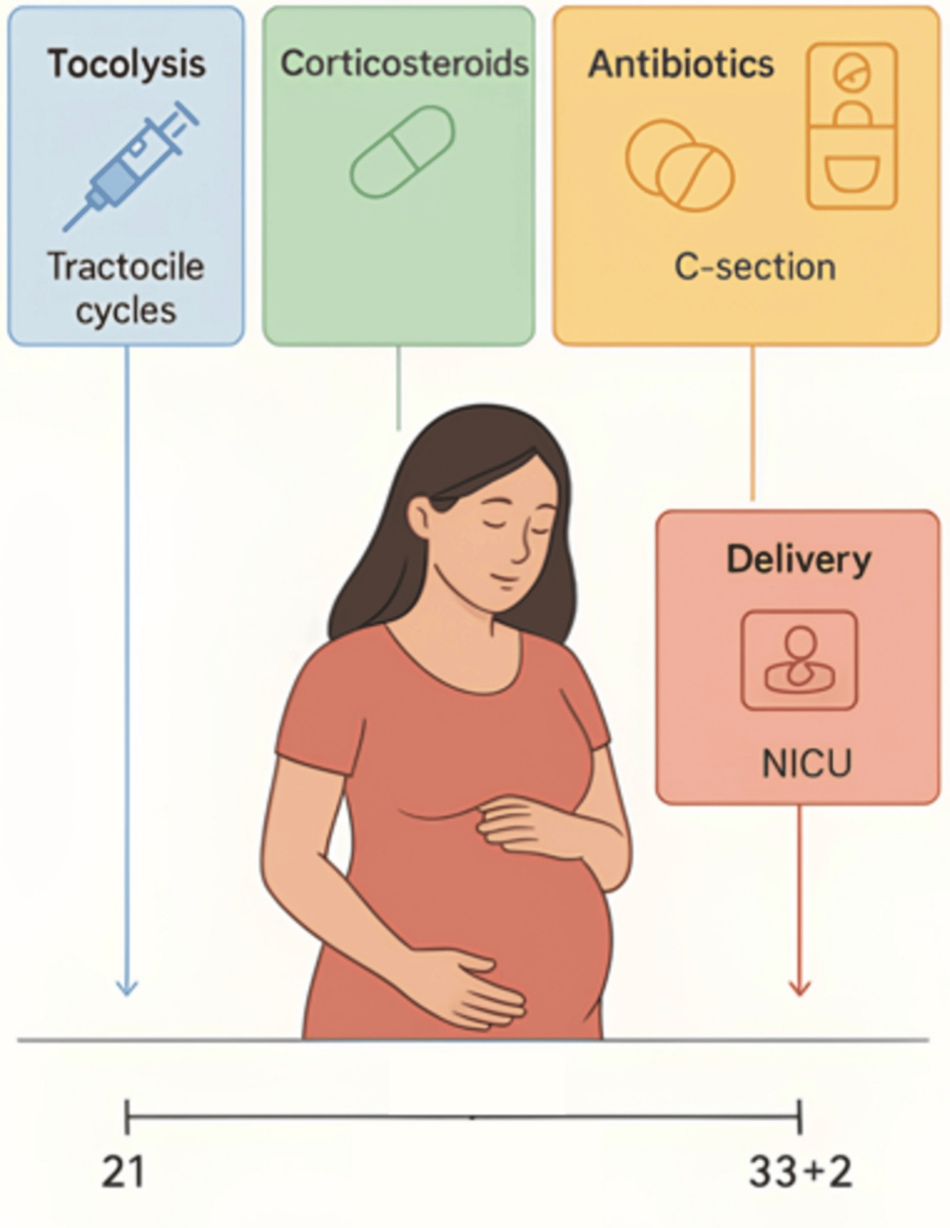
Course of a triplet pregnancy with PPROM Timeline of management during the course of the triplet pregnancy. The figure illustrates the gestational age at which key interventions were undertaken. Tocolysis with Tractocile was initiated at 21–22 weeks, followed by administration of corticosteroids and antibiotics during the subsequent weeks. The pregnancy was ultimately delivered by cesarean section at 33 weeks and two days, with admission of the newborns to the neonatal intensive care unit (NICU). The figure is created by the authors of this study. PPROM: preterm premature rupture of membranes

On September 5, 2025 (33 weeks and two days), an urgent cesarean section was performed for non-reassuring FHRs. Two preterm infants were delivered with favorable Apgar scores. Maternal postoperative recovery was uncomplicated, and both neonates had stable NICU courses (Figure 1).

## Discussion

Multifetal pregnancies are associated with a wide spectrum of maternal and neonatal complications, with preterm premature rupture of membranes (PPROM) and prelabor rupture of membranes (PROM) representing two of the most challenging scenarios. PPROM is defined as rupture of membranes before 37 weeks of gestation, whereas PROM refers to rupture at or after 37 weeks. In multiple gestations, PPROM and PROM carry heightened risks of prematurity, intrauterine infection, oligohydramnios-related complications, and adverse neurodevelopmental outcomes, making management particularly complex [6,7]. Compared with singletons, multifetal pregnancies are more vulnerable to neurologic morbidity, including intraventricular hemorrhage (IVH) and cerebral palsy, largely due to the higher rates of extreme prematurity [8,9]. Ischemic events such as placental abruption and cord accidents also occur more frequently in multifetal pregnancies, further amplifying fetal risk [10].

Expectant management is frequently attempted in multifetal gestations when PPROM occurs before viability or in the peri-viable period, particularly when one fetus demises while others remain viable. Prognosis is strongly influenced by gestational age at rupture, duration of latency, and the presence of infection or placental complications. While conditions such as twin-twin transfusion syndrome (TTTS) are unique to monochorionic gestations, similar placental and hemodynamic considerations influence outcomes in higher-order multifetal pregnancies as well [11,12]. Disseminated intravascular coagulation (DIC), although rare, remains a recognized maternal risk following intrauterine fetal demise; with careful serial monitoring of coagulation parameters, continuation of pregnancy is generally safe [13].

The timing of delivery following PPROM remains one of the most debated aspects of management. In both singleton and multifetal pregnancies, absolute indications for immediate delivery include non-reassuring fetal status, clinical chorioamnionitis, or significant placental abruption [14]. In the absence of these conditions, management is primarily guided by gestational age. Most guidelines recommend delivery at ≥34 weeks, with expectant management prior to this threshold when maternal and fetal conditions are stable [15]. Hospitalization with close maternal and fetal surveillance is typically recommended, although optimal monitoring frequency is not standardized. Serial maternal laboratory parameters and fetal growth trends are therefore often interpreted based on overall clinical patterns rather than isolated values.

Tocolysis in the setting of PPROM highlights a major divergence between singleton- and multifetal-based evidence. In singleton pregnancies, meta-analyses have demonstrated that tocolytics may prolong latency but often without significant improvement in neonatal survival and with a potential increase in maternal complications [16]. Consequently, organizations such as the American College of Obstetricians and Gynecologists (ACOG), the Royal College of Obstetricians and Gynaecologists (RCOG), and the World Health Organization (WHO) advise against routine tocolysis in PROM, whereas the Collège National des Gynécologues et Obstétriciens Français (CNGOF) remains neutral due to insufficient evidence. In multifetal gestations, however, available data are sparse and heterogeneous, and management decisions are often individualized.

The present case illustrates how short-term, carefully selected tocolysis may offer meaningful benefit in multifetal pregnancies by prolonging gestation sufficiently to improve neonatal outcomes. When tocolysis is considered, nifedipine is generally recommended as first-line therapy, with atosiban as an alternative; betamimetics are avoided due to cardiovascular toxicity [16]. Atosiban acts as a competitive antagonist of oxytocin and vasopressin V1a receptors, inhibiting the phospholipase C-inositol triphosphate signaling pathway and reducing intracellular calcium release, thereby suppressing myometrial contractility [17]. In this case, its use allowed uterine stabilization following selective fetal loss without precipitating infection or immediate delivery of the remaining fetuses.

By contrast, antenatal corticosteroids are universally recommended in both singleton and multifetal pregnancies, with strong evidence supporting reductions in neonatal mortality, respiratory distress syndrome (RDS), IVH, and necrotizing enterocolitis (NEC), without increasing infection risk [14]. Antibiotic therapy is likewise essential during expectant management, as it prolongs latency and reduces infectious morbidity [14].

Despite these interventions, outcomes in multifetal pregnancies complicated by early PPROM remain poorer than those observed in singletons. Survival rates following PPROM before 26 weeks of gestation are estimated at 40-50%, with substantial risks of pulmonary hypoplasia, sepsis, and neurologic injury [18]. Latency duration tends to be shorter in multifetal gestations, contributing significantly to adverse outcomes. Notably, evidence suggests that in multifetal pregnancies, chorioamnionitis may more commonly arise as a consequence rather than a cause of membrane rupture, differing from patterns observed in singleton pregnancies [18].

The incidence of PROM increases with fetal number, occurring approximately three times more frequently in twins than in singletons and at even higher rates in triplet gestations. Complications include cord prolapse, cord compression due to oligohydramnios, placental abruption, and maternal and neonatal sepsis. When PROM or PPROM affects a single sac in a multifetal pregnancy, clinicians must carefully balance the risks of expectant management for the remaining fetuses against maternal infectious morbidity, underscoring the complexity of decision-making in such cases [18].

Additional perinatal factors also influence outcomes. In pregnancies conceived through ART, spontaneous fetal reduction is relatively common in higher-order gestations and is often preceded by vaginal bleeding. Furthermore, chorionicity plays a critical role in prognosis, with dichorionic and trichorionic pregnancies generally associated with lower risks of inter-fetal complications compared with monochorionic gestations [19].

Similar cases of multifetal pregnancies complicated by PPROM and selective fetal loss have been described in the literature, demonstrating that individualized expectant management with close maternal surveillance can result in survival of remaining fetuses in carefully selected cases [15,18]. Neonatal outcomes in the present case included favorable birth weights, appropriate postnatal growth, and stable short-term NICU courses without major complications, supporting the effectiveness of the management strategy employed.

Taken together, although the prognosis of multifetal pregnancies complicated by PPROM remains guarded, particularly at earlier gestational ages, this case supports the role of individualized, closely monitored expectant management. Current best practice includes antenatal corticosteroids, antibiotic therapy, and vigilant surveillance prior to 34 weeks, with delivery thereafter. While routine tocolysis is discouraged based largely on singleton data, selective use in multifetal gestations may provide a critical latency window for fetal maturation, as demonstrated in this triplet pregnancy.

## Conclusions

This case demonstrates that selective PPROM in multifetal pregnancies can be managed successfully with individualized, closely monitored care. In the presented TCTA triplet pregnancy, one fetus experienced PPROM and cord prolapse while the other two remained viable. Conservative management, including short-term tocolysis with atosiban, antenatal corticosteroids, antibiotics, and intensive maternal-fetal surveillance, allowed prolongation of gestation and resulted in the survival of two neonates. The case highlights that tailored interventions, applied cautiously in higher-order multiples, can achieve favorable neonatal outcomes even when standard guidelines derived from singleton pregnancies would typically recommend immediate delivery.

This case further illustrates the potential role of selective tocolysis in stabilizing remaining fetuses, the importance of multidisciplinary coordination, and the need for vigilant monitoring of maternal and fetal status. Management decisions in multifetal gestations should be individualized, balancing the risks of infection and preterm birth against the potential benefits of prolonging gestation. Finally, it emphasizes the need for prospective studies to establish evidence-based protocols for selective PPROM in multifetal pregnancies.
